# COVID-19 Vaccination Rates in a Cohort Study of Patients With Mental Illness in Residential and Community Care

**DOI:** 10.3389/fpsyt.2021.805528

**Published:** 2021-12-16

**Authors:** Victor Mazereel, Tom Vanbrabant, Franciska Desplenter, Johan Detraux, Livia De Picker, Erik Thys, Ken Popelier, Marc De Hert

**Affiliations:** ^1^University Psychiatric Center KU Leuven, Leuven, Belgium; ^2^Department of Biomedical Sciences, Center for Clinical Psychiatry, KU Leuven, Leuven, Belgium; ^3^Department of Pharmaceutical and Pharmacological Sciences, Clinical Pharmacology and Pharmacotherapy, KU Leuven, Leuven, Belgium; ^4^Scientific Initiative for Neuropsychiatric and Pyschopharmacological Studies, University Psychiatric Hospital Campus Duffel, Duffel, Belgium; ^5^Psycho-Sociaal Centrum St.-Alexius-Elsene Vzw, Ixelles, Belgium; ^6^Antwerp Health Law and Ethics Chair – AHLEC University Antwerpen, Antwerp, Belgium

**Keywords:** severe mental illness (SMI), COVID-19, vaccination, cohort study, vaccine uptake

## Abstract

**Background:** Patients with mental illness are at increased risk for COVID-19-related morbidity and mortality. Vaccination against COVID-19 is important to prevent or mitigate these negative consequences. However, concerns have been raised over vaccination rates in these patients.

**Methods:** We retrospectively examined vaccine uptake in a large sample of Belgian patients admitted to or residing in a university psychiatric hospital or community mental health care setting between 29th of March 2021 and 30th of September 2021 in the Flanders Region. All patients were offered vaccination. Descriptive statistics were used to analyse the data. Logistic regression was used to examine factors associated with vaccine uptake.

**Results:** 2,105 patients were included in the sample, of which 1,931 agreed to be vaccinated, corresponding with a total vaccination rate of 91.7%. Logistic regression showed an effect of the diagnosis “other disorders” (OR = 0.08, CI = 0.005–0.45), age (OR = 1.03, CI = 1.02–1.04) and residing in the psychosocial care center (OR = 0.50, CI = 0.32–0.80) on vaccination status.

**Conclusion:** Vaccine uptake among people with mental illness is high and comparable to the general population, when implementing a targeted vaccination program.

## Introduction

Vaccination programs have led to the successful control or even eradication of transmissible diseases such as polio, smallpox or the measles in the past (1). In the context of the ongoing COVID-19 pandemic, vaccination programs provide an essential measure to reduce hospital admission and mortality. Due to the initial scarcity of vaccines, some subgroups of the population were prioritized above others based on the risk of infection or COVID-19-related morbidity and mortality (2). People suffering from severe mental illness also should be prioritized in these allocation strategies for a number of reasons. Firstly, severe mental illness is correlated with a wide array of physical problems such as diabetes, hypertension, chronic obstructive pulmonary disease and obesity, which each on its own increase the risk of severe COVID-19 (3–6). Secondly, severe mental illness is positively correlated with other risk factors, such as social-economic deprivation, institutionalization, less adequate health-seeking behaviors and lack of access to appropriate health care, further substantiating this group as vulnerable in the current pandemic (3, 6, 7). Lastly, several recently published meta-analyses and systematic reviews have shown several mental health disorders to be associated with increased COVID-19-related hospital admissions and mortality, even when adjusted for the above-mentioned factors (8–10). This indicates that mental health disorder- and treatment-related issues (i.e., immunological disturbances, accelerated aging, psychotropic medication) may further increase the risk of COVID-19-related mortality in these people (10). However, vaccination strategies have often overlooked this vulnerable population (11).

To reduce COVID-19-related hospital admissions, and thus to be able to release the strenuous socio-economic measures taken to reduce the health impact of the pandemic, it is essential that vaccine coverage in the population is sufficiently high. Vaccination coverage depends on many factors including vaccine production, distribution and uptake in the population (7). Vaccine uptake proves to be an important but amenable barrier in reaching adequate vaccination coverage. In this sense, both the attitude of the population toward a vaccine and the removal of any practical barriers against vaccination are essential to address. Vaccine hesitancy, defined as the delayed acceptance or refusal despite available vaccine services, is associated to factors such as trust in the vaccine and/or the caregiver, knowledge of vaccine safety, knowledge of why the vaccine is necessary and easily having access to the vaccine (7, 12, 13). It is therefore essential to examine in which populations vaccine hesitancy is more prevalent and to identify the determinants being able to effectively counter this.

According to the 2019 National Survey on Drug Use and Health, about one in five adults in the US are suffering from a mental health disorder at any given time (14). This group therefore is an important fraction of the total population. Only granting priority access to people with a mental health disorder in national vaccination strategies will not be sufficient, as a significant COVID-19 vaccination gap seems to exist between these patients and the general population. A recent longitudinal cohort study conducted in Israel found lower COVID-19 vaccination rates in a population of people with schizophrenia when compared to the general population (15), despite having been granted early universal or priority access to SARS-CoV-2 vaccination. These study results are similar to these of an earlier study conducted in 2013 which found lower influenza vaccination rates among patients with mental illness (16). However, other studies have shown comparable willingness to be vaccinated or vaccination rates between the general population and in people with mental illness (17, 18). Nevertheless, targeted vaccination programs can substantially increase vaccination uptake in people with mental illness (19). One possibility would be to actively reach out and offer vaccination to patients with mental illness in mental health clinics and community care services (20).

In a previous study we assessed vaccine uptake in hospitalized patients from a single large university hospital in Belgium and compared it to the national vaccination uptake rates (18). In the current study, we provide further data on vaccination rates in both residential and community care services in Belgium.

## Methods

### Sample

We retrospectively gathered data from all patients admitted to or residing in the University Psychiatric Center KU Leuven, a university psychiatric hospital and community mental health services in Belgium, between the start of the vaccination program on 29th of March 2021 and 30th of September 2021. The community mental health services consisted of a night hospital in Brussels (Psychosocial Center) (21, 22), and a sheltered housing setting and ambulatory care. In a targeted vaccination program, all patients older than 12 years were offered the opportunity to be vaccinated by their healthcare provider when admitted to or residing in the care setting as part of the Belgian national vaccination strategy. When patients had concerns about vaccination, clear information about individual risks and benefits of the vaccine was given. In addition, the governmental vaccination program invited people depending on priority level. Some people might have already been offered the opportunity for vaccination before they were admitted to the hospital Age, sex, main diagnostic code according to DSM-IV at admission, ward or team to which the patient was admitted, vaccination status and type of vaccination were recorded. Diagnostic codes were divided into 11 categories: cognitive, psychotic, bipolar, depressive, developmental, anxiety, personality, substance use, eating, adjustment, and other disorders which consisted most of “deferred diagnosis” and psychosocial problems.

### Statistical Analysis

Descriptive statistics were used to analyze the data. Only patients with known vaccination status were included. Duplicate data were removed. Primary outcome was vaccination status (vaccinated vs. not-vaccinated/refused). Binary logistic regression was used to evaluate whether type of disorder, level of care, age and sex were associated with vaccination status. The reference group in the logistic regression consisted of males with a cognitive disorder from the University Psychiatric Center due to coding of the variables. Significance level was set at α = 0.05.

## Results

**Figure 1:**
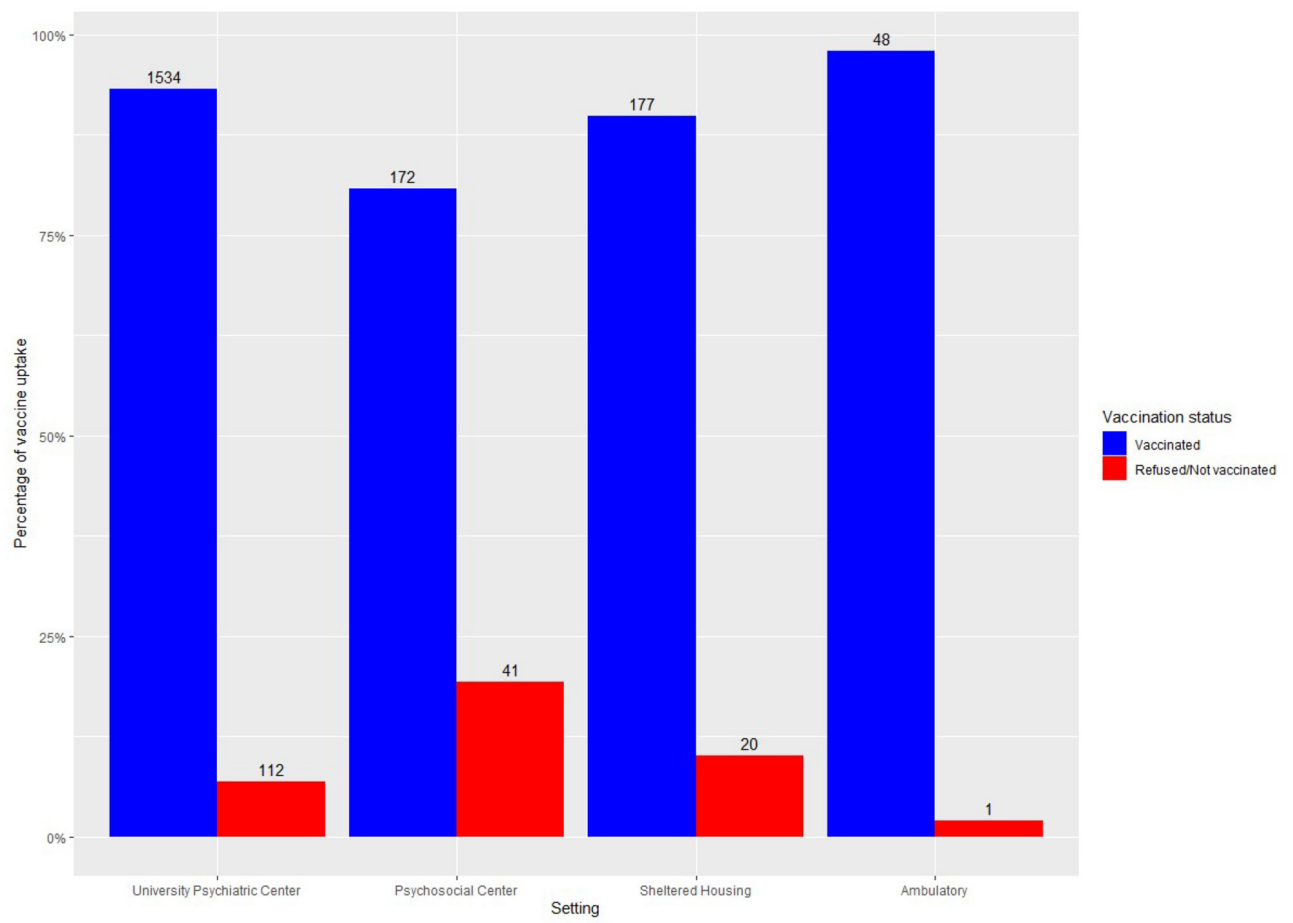
Vaccine uptake in a university psychiatric hospital and in community mental health services.

**Figure 2:**
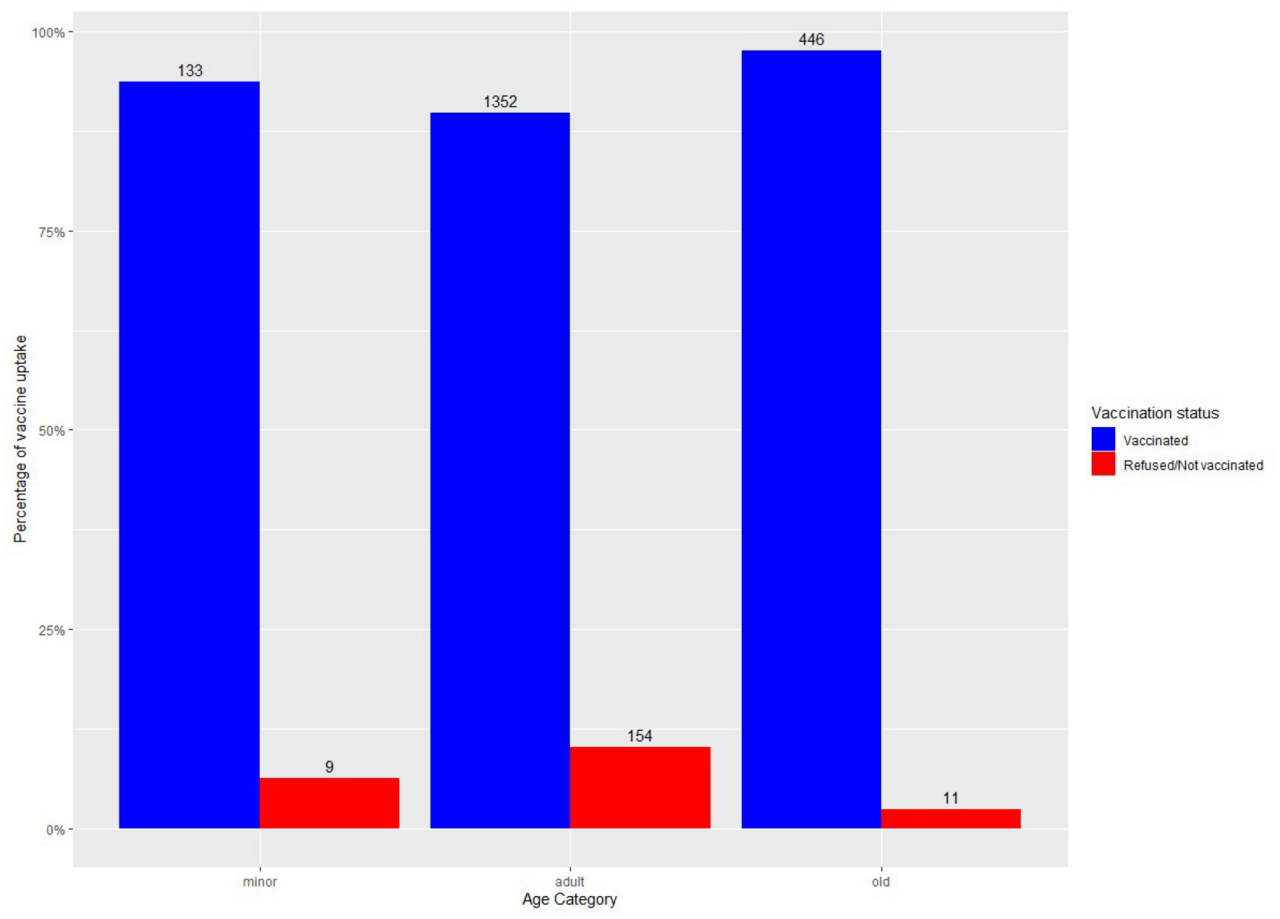
Vaccine uptake per age category.

Two thousand one hundred and five patients were offered COVID-19 vaccination, of which 1,931 accepted vaccination, corresponding with a vaccination rate of 91.7%. Results are shown in Table 1. Logistic regression showed an effect of the diagnosis “other disorders” (OR = 0.10, CI = 0.005–0.53), age (OR = 1.03, CI = 1.02–1.04) and residing in the psychosocial center (OR = 0.48, CI = 0.30–0.77) on vaccination status. Percentage of vaccination rate per care setting and per age category are shown in Figures 1, 2, respectively.

**Table 1:** Demographic characteristics.

**Characteristic**	**Value**
*Age*	46.9 (SD = 20.6)
*Gender*	41.3% male
*Diagnosis (n = 2,105)*	
Cognitive disorder	157 (7.5%)
Psychotic disorder	540 (25.7%)
Bipolar disorder	151 (7.2%)
Depressive disorder	240 (11.4%)
Developmental disorder	45 (2.1%)
Anxiety disorder	153 (7.3%)
Personality disorder	175 (8.3%)
Substance use disorder	103 (5.0%)
Eating disorder	84 (4.0%)
Adjustment disorder	248 (11.8%)
Other	209 (9.9%)
*Vaccine status (n = 2,105)*	
Fully	1,782 (84.6%)
Partially	148 (7.0%)
Refused	174 (8.3%)
*Vaccine Type (n = 1,931)*	
Moderna	854 (44.2%)
Pfizer	843 (43.7%)
AstraZeneca	176 (9.1%)
Johnson & Johnson	56 (2.9%)

## Discussion

Adequate vaccination coverage against COVID-19 will be an important step to combat this pandemic. Concerns have been raised that people with mental illness would be less willing or less able to be vaccinated. We have previously shown that these concerns are undeserved when a targeted vaccination program is set up in inpatient settings (18). Here we extend our previous findings to community health care services and show that the actual vaccination rate is very high (91.7%) when a targeted vaccination program is set up and people with mental illness are offered the chance to be vaccinated as part of routine clinical care (23).

For the European region median cumulative vaccine uptake from September 27 to October 3 2021 for a first dose was 69.6% (range 15.7–98%) for adults over 18 years, 75.7% (range 0–99.6%) for adults over 60 years and 11.2% (range 0–31.9%) for young people under 18 years (24). These figures show that vaccine uptake varies considerably. Thus, it can be expected that vaccine uptake for people with mental illness will also vary considerably internationally. Vaccination rates in Belgium are region-dependent. Although the vaccination coverage rate in Wallonia, the German-speaking region, and particularly in Brussels Region are lower, vaccination rate on 30th of September 2021 in Flanders, Belgium was 91.9% for adults, 95.9% for people older than 65 and 84% for minors between 12 and 18 (25). Our results are consistent with these vaccination rates, with an additional amount of young people that had their vaccination.

Logistic regression showed an effect of age with older people being more likely to be vaccinated. This is not surprising as they also experience a higher risk for hospitalization and mortality (26, 27). They were also among the first being able to receive a vaccine since prioritization of vaccination included people older than 65 in Belgium. However, when looking at vaccination rate per age group (Figure 2) we see in our sample that minors are also very willing to be vaccinated. Although they experience less of an increased risk for negative outcomes of COVID-19, they can also contribute to collective immunity of the population, and as such play a decisive role in combatting this pandemic. Recent research has shown the importance of vaccination of children and adolescents in reducing transmission and disease burden of COVID-19 (28).

We did not find an effect of diagnosis on vaccination uptake, although the category of ‘other diagnoses’ was less likely to be vaccinated. This category mostly consisted of cases of deferred diagnosis (*n* = 149) or psychosocial problems (*n* = 14). We hypothesize this was confounded by a higher representation of people from the psychosocial community center (40.6%) in this diagnostic category than in the full sample (where they represent 10.1%).

People from the psychosocial community center were less likely to be vaccinated. This is likely due to the geographical location of the community center which lies in the Brussel Region. Vaccine uptake in Brussels is lower than in Flanders (67.2% of adults have had their first dose, 82% of elderly above 65 years, 33% of minors between 12-18 years, data for 30th of September 2021). This provides evidence for the argument that vaccination rates in people with mental illness are similar to those of the general population.

Previous systematic reviews and meta-analyses have shown an elevated risk for COVID-19-related morbidity and mortality in people with mental disorders (8–10, 29). This is added on top of an already decreased life span of about 20 years for people suffering from severe mental illness (30, 31). Different factors attribute to this increased risk, implicating both biological and socio-economic pathways. The surest way to decrease this excess risk of COVID-19-related infection, morbidity and mortality is prevention by vaccination. As we have argued previously (3, 6) this knowledge urges us to prioritize and target patients with (severe) psychiatric disorders for COVID-19 vaccination and to develop intentional vaccine delivery strategies for these people, including a booster vaccine dose. National guidelines are slowly picking this up (11) and recently the United States Center for Disease Control has also added mental disorders to the list of high-risk groups (32).

Concerns have been raised in previous research as to the acceptance and uptake of vaccines in people with psychiatric disorders. One study found a slightly lower willingness to be vaccinated when compared to the general population, although the difference was rather small (17). In a Chinese survey only 78.7% of patients and family members intended to receive a vaccine (33). Another small study in a medium security psychiatric hospital showed that 20% of patients declined consent to be vaccinated (34). Our results show that with a targeted vaccination program the majority of patients residing in a psychiatric hospital or attended to in community psychiatric services accept and get the vaccine and that they are just as willing and accepting as the general population. However, we recognize that vaccine acceptance or hesitancy may be highly regionally variable (35–37). In Belgium, vaccine willingness and acceptance are generally high, especially in the Flanders region, attested by the high vaccine uptake in the general population. Large parts of the world are unfortunately still waiting for the chance at a first vaccine shot. To ensure maximal vaccine uptake healthcare providers and policy makers should provide enough information on vaccine safety, individual risk assessment and explicitly recommend vaccination as support from healthcare providers increases chance of vaccination (33, 34, 36, 38, 39).

### Limitations

Strengths of our study are the large sample size and the chance for vaccination for each patient admitted to the hospital or community care service. We were able to include both patients from a hospital setting and from a community mental health service or ambulatory care service. The community mental health service did focus more on patients with severe mental illness. Future research, however, should examine vaccine uptake in other ambulatory care settings as well. Another limitation is the generally high acceptance of vaccination in Belgium. This might be drastically different in other countries. Nevertheless, our results also show that patients with psychiatric disorders are just as likely to be vaccinated as the general population. Therefore, one can argue that by increasing vaccine acceptance in the general population, one will also increase acceptance in people with psychiatric disorders.

## Conclusion

To release restrictive measures within mental health care settings and combat the COVID-19 pandemic vaccination of psychiatric staff and patients is of primary importance and will depend on vaccine acceptance and uptake. There have been some concerns that people with psychiatric disorders would be less likely to receive vaccination. Our results show that people with psychiatric disorders are just as likely to accept and receive vaccination as the general population. Type of disorder does not seem to play a role in the vaccine uptake rate. We encourage healthcare providers and policy makers to prioritize vaccination for this vulnerable group. Focusing on safety profile, providing enough information on individual risk and explicitly recommending vaccination are ways to increase vaccine uptake.

## Data Availability Statement

The raw data supporting the conclusions of this article will be made available by the authors, without undue reservation.

## Ethics Statement

Ethical review and approval was not required for the study on human participants in accordance with the local legislation and institutional requirements. Written informed consent from the participants' legal guardian/next of kin was not required to participate in this study in accordance with the national legislation and the institutional requirements.

## Author Contributions

VM and TV wrote the manuscript. VM, TV, FD, KP, and MD gathered the data. VM performed the statistical analyses. FD, JD, ET, LDP, and MD revised the manuscript. All authors approved the final manuscript.

## Conflict of Interest

The authors declare that the research was conducted in the absence of any commercial or financial relationships that could be construed as a potential conflict of interest.

## Publisher's Note

All claims expressed in this article are solely those of the authors and do not necessarily represent those of their affiliated organizations, or those of the publisher, the editors and the reviewers. Any product that may be evaluated in this article, or claim that may be made by its manufacturer, is not guaranteed or endorsed by the publisher.
